# Mycoviruses: Environmental Variables, Vector-Mediated Transmission and Use as a Biocontrol

**DOI:** 10.3390/v18030300

**Published:** 2026-02-28

**Authors:** Glenda Coromoto Velasquez Serra, Patricia Elizabeth Molleda

**Affiliations:** 1 Medicine Career, Faculty of Medical Sciences, University of Guayaquil, Guayaquil 090510, Ecuador; 2 Faculty of Health Science and Human Development, Ecotec Technological University, Km. 13.5 Samborondon, Guayaquil 092302, Ecuador; pmolleda@ecotec.edu.ec

**Keywords:** mycovirus, arthropods, genome, disease

## Abstract

Mycoviruses, or fungal viruses, are widespread throughout the fungal kingdom. This study aimed to identify environmental factors associated with mycoviruses, explore their transmission mechanisms, including vector-mediated transmission, and assess their potential practical applications. A systematic, retrospective, and cross-sectional bibliographic review was conducted. These viruses are closely linked to their fungal hosts, thus developing a symbiotic relationship. Among environmental variables, temperature appears to play a more significant role in limiting fungal virulence than other factors, such as relative humidity or ultraviolet radiation. Most mycoviruses are generally asymptomatic RNA viruses, rarely affecting the host’s phenotype, and are transmitted intracellularly, primarily through vertical transmission via spores or horizontally through hyphal anastomosis; therefore, their life cycle typically lacks an extracellular phase. Mycoviruses remain understudied, particularly their role in vector-borne transmission and their influence on pathogen structure and behavior. Transmission can be persistent, where the virus replicates in insect vectors and is passed to offspring via infected eggs or feces, or non-persistent and indirect, facilitated by vectors without replication. Understanding the biology and ecology of mycoviruses is essential for advancing their potential use in the biocontrol of pathogens, including phytopathogens, representing a promising area of applied research.

## 1. Introduction

Mycoviruses, or fungal viruses, are widespread throughout the fungal kingdom [1]. Mycoviruses biologically control pathogenic fungi, which means they have the ability to convert them into beneficial endophytes, or they may reduce fungal agents’ effectiveness [1,2,3,4]. Mycovirus that elicit hypovirulences have been identified in both human and plant pathogenic fungi [5,6,7].

Historically, mycoviruses were found in diseased *Agaricus bisporus* mushrooms and *Aspergillus* spp. ascomycete over 50 years ago [5,6]. Mycovirus were also identified in the *Penicillium stolonifer* (ascomycete), causing interferon stimulation in mammals [7].

They are known to associate with most major taxonomic groups of fungi, including *Ascomycota*, *Basidiomycota*, *Chytridiomycota*, *Zygomycota*, and *Neocallimastigomycota* [7]. However, the last updated report of the international committee on Taxonomy viruses (ICTV) on virus taxonomy lists more than 90 mycovirus species spanning over 47 viral families, and one new currently recognized genus is *Botybirnavirus*; however, 20% are not assigned to a genus or, in some cases, are not even assigned to a family [1,8,9,10,11].

Currently, Mycoviruses are allocated to six phyla: *Negarnaviricota*, *Duplornaviricota*, *Kitrinoviricota*, *Pisuviricota*, *Lenarviricota*, and *Artverviricota*, within three kingdoms: *Shotokuvirae*, *Orthornavirae* and *Pararnavirae*. They also have broad similarities in the architecture and genomic organization of many fungal and plant RNA viruses [4,12,13].

Mycoviruses are molecularly defined as extrachromosomal nucleic acid chains replicated by a fungal host. There is a wide range of genome sequences of naked or packaged DNA and RNA molecules, but the majority of known mycoviruses have double-stranded RNA (dsRNA) or positive-sense single-stranded RNA (+ssRNA) genomes. A minority are negative-sense (−) ssRNA or DNA viruses [1]. Currently, viruses belonging to 47 families are reported, (19 officially recognized families and 1 unclassified genus), including “+ssRNA” “ssRNA”, “double-stranded RNA”; “dsRNA”; and “ssDNA” viruses [14].

Myers and James (2022) [15] highlight that approximately 200 mycoviruses have been described, and that a few hundred more have been characterized and detected in almost all fungal clades, especially in the *Pezizomycotina* a group of filamentous fungi with the greatest diversity of species, such as *Aspergillus*, some phytopathogenic fungi, and some other detected endophytes. Fungi such as *Beauveria bassiana* have been reported to harbor up to 11 putative mycoviruses, *Agaricus bisporus* 16, *Fusarium poae* 16, and *Fusarium mangiferae* 11. These numbers refer to virus-like sequences identified through metagenomic or transcriptomic approaches and may not all represent functionally validated viruses. Human pathogens such as *Candida albicans* or *Cryptococcus neoformans* have not been reported yet to harbor active viruses [14,15,16,17,18].

*Aspergillus* is the most studied fungal genus due to its agricultural, clinical and economic importance. The first mycovirus was reported in this fungal genus in 1970. To date, viral particles have been found in strains belonging to *Aspergillus nigri*, *Aspergillus clavati*, *Aspergillus circumdatus*, *Aspergillus fumigatus*, and *Aspergillus flavus* [17,19].

Since 1986 [17,18], the interaction between mycoviruses and mycotoxigenic fungi has been debated, specially aflatoxigenic species, as it was found that some viruses have effects on the biosynthesis of aflatoxins, especially those produced by *Aspergillus flavus*. This fungus has a high incidence on agroecosystems, especially cereal crops, with possible repercussions for human and animal health due to aflatoxin contamination of feed and other food products; therefore, it is important to further investigate the interaction between *Aspergillus flavus* and mycoviruses [19].

According to Schmidt et al. (1986) [18], studies carried out on *A. flavus*, specifically on the *NRRL5565* strain, demonstrated that the production of carcinogenic aflatoxin was inhibited due to the presence of mycoviruses. Researchers such as Silva et al. (2001) [19] describe in their research that the *NRRL5940* strain of the same fungus produces aflatoxins with fewer viral particles. The presence of mycotoxins in agricultural crops and the fact that they are consumed directly and indirectly in meat and dairy products represents a problem of medical and economical importance for developed countries; therefore, controlling the production of aflatoxins through the use of mycoviruses could be beneficial for human beings [2,20,21,22,23].

As for *Aspergillus fumigatus*, it is a fungus of clinical importance that causes aspergillosis. The hypo-virulent and hypervirulent effects of viruses that use this fungus as a host should be studied. Studies should also be carried out on mycoviruses in clinical samples and the relationships between the parameters of clinical pathologies and patient survival, since aspergillosis occurs in patients with hematological malignancy or those undergoing an organ transplant [21,23]. Mycoviruses in *Aspergillus* spp. have been studied more frequently than in any other fungal genus. The mycoviruses that were found are *Partitiviridae*, *Chrysoviridae*, and *Totiviridae*, and new families have appeared, such as *Polymycoviridae*, *Alternaviridae*, and *Yadokariviridae* [20].

The most notable characteristic of mycoviruses is that they lack an extracellular route of infection and are only transmitted intracellularly through hyphal anastomosis (horizontal transmission) or during sporogenesis (vertical transmission) [1,21,22]. The replication, transcription, and translation of mycoviruses depend on their host; some mycoviruses can reside in the nucleus or mitochondria. For example, the killer virus of *Sacharomices cereviceae*, which replicates in the cytoplasm and has segments in the nucleus, and *Mitoviruses*, which replicates in the fungal host’s mitochondria [15]. Mycoviruses can be observed by electron microscopy but must first be separated by ultracentrifugation. Their size ranges from 50 to 100 nm [17].

Pioneering research was carried out on the filamentous fungus *Chryphonectria parasitica* infected by mycoviruses, which laid the foundations for future studies on hypovirulence damaging other species of economic importance [8]. When the virus is found in a phytopathogenic fungus host, it may cause changes in its physiology, such as reducing its virulence, causing hypovirulence. This hypovirulence, caused by the presence of mycovirus in the phytopathogenic fungus, is transmitted through hyphae within the species [1,9,21,24,25,26,27].

It is important to mention that the fungus *Colletotrichum* spp. is a pathogen of great importance from a phytopathological perspective, mainly because it causes anthracnose in several species of plants, specifically tropical and subtropical crops and fruits trees such as mango, avocado, and papaya, and also in legumes and vegetables, causing injuries in plants ranging from the seedling stage to adult trees and damaging any part of the plant that is above the ground, such as stems, leaves, flowers, and fruits [9,26,27,28].

Mycoviruses in the *Partitiviridae* or *Chrysoviridae* family have been reported to have effects on the pathogenic behavior of the fungus *Colletotrichum* spp., causing important physiological changes such as an altered morphology, a reduction in vegetative growth, and reduced conidial production; therefore, conducting research on mycoviruses that can infect phytopathogenic fungi could be an ecological alternative to the harmful effects caused by the use of pesticides or chemical control in plants, which have negative effects on the health of humans and the environment [8].

There is limited research on mycoviruses and missing information about their role in vector-borne diseases and how this may or not affect the structure of the pathogen, with possible repercussions for humans. The lack of appropriate infectivity assays often hindered researchers from reaching a coherent conclusion. In this sense, this research aims to acquire information that can serve as a starting point for new research findings. The objectives of this research are to identify environmental variables associated with mycoviruses, establish transmission mechanisms, and look at the vector-mediated transmission and applicability of mycoviruses.

## 2. Materials and Methods

**Figure 1:**
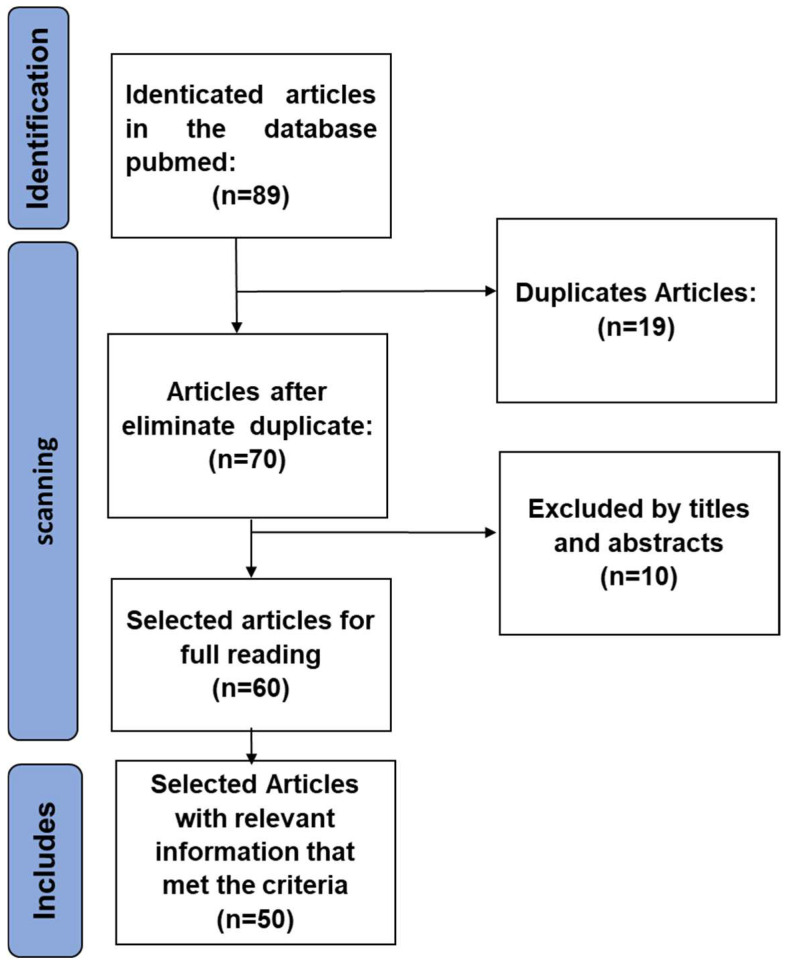
Prisma flow chart showing the search results and screening process. A total of 89 articles were identified that met the inclusion criteria; 70 were selected, of which 10 were excluded, resulting in a total of 50 selected articles with relevant information for this research.

A systematic, retrospective, and cross-sectional bibliographic review to obtain information about mycoviruses was carried out during the period from December 2024 to May 2025. Data were collected from original scientific articles, available in the National Centre for Biotechnology Information (NCBI) PubMed databases, using the following descriptors: “Phytopathogens”, “Mycoviruses”, “Environmental factors”, “Diseases”, “Biocontrol”, combined with AND, OR, and NOT. A total of 89 articles were identified, with 50 articles selected for further study. A summary of the search strategies for the databases is provided in Figure 1. Each article was reviewed for relevance, and those relevant to our topic were included in this review.

Inclusion criteria were as follows: original articles that were peer-reviewed and published in indexed journals, including comparative studies, experimental works, and metanalyses published in English, with no limits regarding year of publication; focused on important topics regarding the general notions of mycoviruses, as well as their most notable characteristics regarding reproduction, environmental variables, transmission mechanisms, internal structures, and vector-mediated transmission; in a field of study focused on mycoviruses and biocontrol strategies; and providing a potential explanation for the symbioses between fungi and viruses. Articles in languages other than English, letters to the editor, editorials, guides, and dissertations were excluded.

## 3. Results and Discussion

The structure of a mycovirus is generally characterized by the following components.

### 3.1. Virion Structure

#### 3.1.1. Viral Genome

This typically consists of nucleic acid, with most genomes being RNA-based. These may include “dsRNA”, “ssRNA+”, or “ssRNA−” [27,28]. This genome, depending on its segmentation, can be either circular or linear, and either monopartite (a single segment) or multipartite (multiple segments) [29,30]. Regarding its size, it generally ranges between 3 and 30 kb, depending on the type of mycovirus [29,30].

#### 3.1.2. Protein Capsid

In this regard, some mycoviruses have capsids of different shapes, composed of capsid proteins [28,29]. Other mycoviruses lack a capsid and exist as ribonucleoprotein complexes within the host fungus [29].

#### 3.1.3. Lipid Envelope

Most mycoviruses lack a lipid membrane, distinguishing them from many animal and plant viruses. Exceptions include mycoviruses associated with lipid vesicles [29].

#### 3.1.4. Viral Proteins

All mycoviruses also encode proteins related to RNA replication, such as RNA-dependent RNA polymerases (RdRp) or reverse transcriptases (RT) for RNA viruses, and replicates (Rep) for DNA viruses include capsid proteins and other enzymes, such as proteases. In more complex mycoviruses, other proteins involved in regulating the infection cycle or interacting with the host may also be present [28,29].

### 3.2. Genome Organization

The genome generally encodes an RdRp, capsid proteins, and, in some cases, additional accessory proteins. Furthermore, the genes are organized in open reading frames (ORFs), which are directly translated within the fungus using its translation machinery [31]. The 5 untranslated region (UTR) of each genomic plus strand is extremely long (1.5 kb), suggesting it is likely to include an internal ribosomal entry site (IRES) for translation initiation (*Megavirnaviridae* dsRNA) [28].

This is because mycoviruses have no extracellular route of transmission, except for a single case involving DNA mycovirus *Sclerotinia sclerotiorum* hypovirulence-associated DNA virus 1 (SsHADV1) [29,30,31,32]. SsHADV1 is not only infectious when applied extracellularly, but it is also capable of reducing virulence in several fungi hosts [32,33]. It remains unclear if extracellular infection of DNA mycoviruses occurs under natural conditions. Further research is needed to understand the prevalence of this phenomenon and whether other DNA mycoviruses can also infect extracellularly. Nevertheless, the capacity for extracellular infection is a major advantage for a potential biocontrol agent, allowing for simpler application and faster spread.

Table 1 shows the family or genus of the most reported mycovirus species in the literature, the type of genetic material they have, fungal host species transmission routes and the function that each one performs in nature or in the fungus it parasitizes. It is highlighted that the mycovirus species *Scletotinia sclerotiorum hypovirus 1 SsHADV-1* belongs to the *Geminivirus* genus unclassified family; it has a circular “ssDNA” genome. Its fungal host is *Sclerotinia sclerotiorum* and it can be transmitted vertically or mediated by mycophagous flies. It is important to mention that mycovirus stimulates plant growth and improves resistance to infection by pathogenic fungus.

The *Alfaflexiviridae* family has two genera, *Sclerodornavirus*, whose mycovirus is *SsDRV Sclerotinia sclerotiorum* RNA virus L with a “+DNA” genome. Its fungal host is *Scletotinia sclerotiorum* and it can be transmitted via ascospores. The other genus is *Botrexvirus*, whose mycovirus is *Botrytix Virus X* with a “+DNA” genome. Its fungal host is *Botrytis cinerea* and it can be transmitted through set genes involved in cell-to-cell and long-distance movement of the plant-infection members of this family.

*Gammaflexiviridae* family include the genus *Mycofexivirus*, whose mycovirus is *Botrytis Virus F* with a “+DNA” genome. Its fungal host is *Botrytis cinerea* and it can be transmitted via ascospores. These mycoviruses are capable of promoting distinct hypovirulence in their phytopathogenic fungal hosts.

The *Hipoviridae* family has only one genus, which is *Hipovirus*, whose individual mycovirus species is *Sclerotinia sclerotiorum hypovirus 1* and *2 (SsHV1/SSVH1/Z-15)*, with “dsRNA” and “+DNA” genomes. Its fungal host is *Scletotinia sclerotiorum* and it can be transmitted via the sclerotia produced by *S. sclerotiorum*, which plays an important role in the vertical transmission of mycovirus in this fungal species, which lacks conidia. These mycoviruses are capable of promoting distinct hypovirulence in their phytopathogenic fungal hosts. Another individual mycovirus species is *Valsa Ceratosperma Hypovirus 1* to *4 (CHV1/CHV2/CHV3/CHV4)*, with a “dsRNA” genome. Its fungal host is *Valsa ceratosperma* and it can be transmitted by hyphal anastomosis. This mycovirus is capable of promoting distinct hypovirulence in its phytopathogenic fungal hosts. *Cryphonectria Hypovirus 1* to *4 (CHV1/CHV2/CHV3/CHV4)* is another individual species of this genus, with “mcRNA+” and “dsRNA” genomes. Its fungal host is *Cryphonectria parasitica* and its infection of fungal mycelium is known to occur only through hyphal contact or by horizontal transmission. This mycovirus is a biological control of chestnut blight in Europe. The individual mycovirus *Fusarium graminearum Hypovirus 1 VgV1 y FgV4*, *FgV2* species belongs to this genus, with an “+RNA” genome. Its fungal host is *Fusarium graminearum* and it can be transmitted by anastomosis hyphal. This mycovirus is capable of promoting distinct hypovirulence in its phytopathogenic fungal hosts.

The *Partiviridae* family has three genus (*Betapartivirus*, *Grammapartivirus* and *Partivirus*). The *Betapartivirus* genus includes individual mycovirus species *Betapartivirus Fusarium poae virus 1* with a “dsRNA” genome. Its fungal host is *Fusarium poae* and infection can occur through hyphal anastomosis, cell division, and sporogenesis. Another individual mycovirus species of this genus is *Sclerotinia sclerotiorum partivirus 1*, with a “dsRNA” genome. Its fungal host is *Sclerotinia sclerotiorum* and it can be horizontally transmitted. The genus *Gammapartivirus* includes two individual mycovirus species: *Penicillium stolonifer virus F* and *Penicillium stoloniferum virus S* with a “dsRNA” genome. Its host is *Penicillium stolonifer* and can be transmitted by hyphal anastomosis, cell division, and sporogenesis of the fungi. *Partivirus* genus has an individual mycovirus species, *HetPV3- and 1*, with a “dsRNA” genome, with the fungal host *Heterobasidion parviporum.* This mycovirus can be transmitted by horizontal transmission or via basidiopores. This fungus is a conifer pathogen and can be controlled with this mycovirus.

The genus *Quadrivirus* belongs to the *Quadriviridae* family; the individual mycovirus species is *Rosellinia necatrix quadrivirus 1* with a “dsRNA” genome. Its fungal host is *Rosellinia necatrix.* It is transmitted by the mycelia and can be used as a virocontrol. The *Megabirnaviridae* family has one genus, *Megavirus*, including the individual mycovirus species *Megabirnavirus 1 (RnMBV1)* with a “dsRNA” genome. Its fungal host is *Rosellinia necatrix* and it can be transmitted via a zinc-mediated method for the horizontal transmission of viruses between strains that are incompatible with the mycelium. The *Reoviridae* family has one genus, *Reovirus*, and four individual mycovirus species (*MycoReovirus*, *MyRV1/MyRV2*, *MyRV3*), all of which have a “dsRNA” genome. *MycoReovirus* fungal host is *Sclerotinia sclerotiorum*, *MyRV1/MyRV2* fungal host is *Cryphonectria parasitica* and both can be transmitted by vertical transmission via conidia and horizontal transmission. The other individual species is *MyRV3* with its host fungus *Rossellinia necatrix*. It can be transmitted by vertical transmission via conidia and horizontal transmission.

The *Barnaviridae* family has one genus, *Barnavirus*, and *Mushroom bacilliform virus* is an individual mycovirus species, with a “dsRNA” genome. Its fungal host is *Agaricus bisporus*. Infection occurs via horizontal transmission via mycelium and possibly vertically by basidiospores. It is important to mention that this virus infects the commonly cultivated button mushroom. The *Totiviridae* family includes *Totivirus* and *Victorivirus*. The *Totivirus* genus has three individual species, *Saccharomyces cerevisiae virus L-A (ScV-L-A)*, with *Saccharomyces cerevisiae* as its host species, and *Ustilago maydis virus H1* and *Ustilago maydis virus P4*, with *Ustilago maydis* as their fungal host. They all have a “dsRNA” genome. The *Victorivirus* genus has one individual species, *Helminthosporium victoriae virus 190S (HvV190S)*, with a “dsRNA” genome. Its fungal host is *Helminthosporium victoriae*. They can be transmitted horizontally via mycelium and possibly vertically by basidiospores.

The *Crysoviridae* family has only one genus, which is Chysovirus, and has two individual mycovirus species, *Penicillium Chrysogenum virus (PcV)*, with the fungal hosts *Penicillium chrysogenum*, and *Cryphonectria nitschke chrysoviridae 1*, an individual mycovirus species whose fungal host is *Chrypthonectria nitschke*; both have the “dsRNA” genome and it is possible to induce a sexual cycle yielding meiotic ascospores. They can be transmitted intracellularly. In the *Narnaviridae* family and the *Narnavirus* genus is a mycovirus individual species with an “+ssRNA” genome. Its fungal hosts are *S. cerevisiae* and *Phytophthora infestans*, which are infected by transmission confined to the cytosol. The *Mycomonegativiridae* family without an assigned genus has the mycovirus species *Sclerotinia sclerotiorum* negative-stranded RNA *virus 1 (SsNSR1)* with an “−RNA” genome. Its fungal host is *Sclerotinia sclerotiotum.*

The *Virgaviridae* family has the Virviviruses genus and has individual mycovirus species *PvLaVVV1* to *PvLaVVV4*, with an “+ssRNA” genome. Its fungal host is *Aspergillus flavus* and its horizontal gene transfer between virus and host has been recognized as an important driving force in the viral evolution. The *Antivirus* family without assigned genus has *Tulasnella ambivirus 1 (MN793991*; *4736 nt)* as an individual mycovirus species with an “ssRNA” genome. Its fungal hosts are *C. parasitica*, *R. solani*; *Armillaria soo*, *H. parviporum*. They can be transmitted by horizontal gene transfer between virus and host, which has been recognized as an important driving force in the viral evolution.

The *Yadokariviridae* family has no assigned genus. The individual mycovirus species is *Yado-kari virus 1 (YkV1)* with an “+ssRNA” genome. Its fungal host is *Rosellinia necatrix*. They can be transmitted by horizontal gene transfer between the virus and the host, which is an important driving force in the viral evolution. The *Polimicoviridae* family has the *Polymicovirus* genus and two individual species. One is *Aspergillus fumigatus tetramycovirus 1 (AfuPmV1)* with an “+ssRNA” genome. Its fungal host is *Aspergillus fumigatus* and its transmission mycelia are associated with one of the virally encoded proteins. The other individual species is *Colleotrichum camellia filamentous virus 1 (CcFV1)* with a “dsRNA” genome. Its fungal host is *Colletotrichum camelliae*. The virus can be transmitted vertically by sexual spore and horizontally by hyphas anastomosis transfer of the virus. A morphological characterization of all mycoviruses is necessary.

### 3.3. Environmental Variables Associated with Mycoviruses

There is limited information on the ecological and environmental importance of mycoviruses, especially those present in entomopathogenic ascomycetes. Environmental variables are generally associated with the fungal host of the existing mycovirus and their symbiosis. Authors such as Rueda-Maillo et al. (2024) [39,40], in their work on how a mycoviral infection enhances the virulence and ecological suitability of the fungus *Beauveria bassiana*, subjected to biotic and abiotic environmental stress, consider that climatic factors such as temperature affect the success of entomopathogenic ascomycetes in pest control, since this factor limits fungal virulence more than other factors, such as relative humidity and ultraviolet radiation. It is also important to note that the climatic factors affecting organisms that are in antagonistic relationships with other microorganisms, such as a virus, tend to exhaust and inactivate them, especially in cases of entomopathogenic ascomycetes, whose habitat is epigeal and hypogeal.

Studies carried out with infected the fungus *Beauveria bassiana* showed that when grown at temperatures of 10 to 35 °C, its growth and germination rate increased from 0.2 to 21.5% [38,39]. Other studies carried out with the fungus *Curvularia protuberata* demonstrated that this species, in symbiosis with other viruses, provided thermal protection to this fungus, allowing for an increase in growth at 30 °C and managing to survive at temperatures of 38 °C [40]. Other authors mention that a strain of *Tolypocladium cylindrosporum* infected with viruses showed greater growth at 30 °C [35,36,37].

The wild strain of the fungus *Beauveria bassiana* showed a wider temperature range for growth and germination. This fungus presented an increase in the germination rate in the infected fungal mycovirus strain compared to a mycovirus-free strain. Temperature ranges evaluated were between 10 °C and 35 °C. Despite the lack of existing data on how temperature influences the ecological adaptation of mycoviruses and their influence on the host fungus, it has been determined that a wider temperature range causes slower growth. This characteristic was also observed in *Aspergillus* spp. and in *Rhizoctonia solani*, with the mycovirus-free stain showing more growth at temperatures between 20 and 28 °C [38,39,40,41,42,43].

The study conducted by Rueda-Maillo et al. (2024) [40] demonstrated, for the first time, that mycovirus infection promotes drought tolerance in the fungus host *Beauveria bassiana*, showing a germination rate of 2.6% to 0.9% compared to no germination for the virus-free strain. It is important to mention that osmotic stress significantly affects the growth of the fungus but benefits the liberation of the virus. This tolerance to osmotic stress was also observed in the virus-infected fungus *Cryphonectria parasitica* [32,38,40,41,42]. By utilizing the *Bauveria bassiana* strain naturally infected with two mycoviruses, *Beauberia bassiana partitivirus 2 (BpPV2)* and *Beauberia bassiana polymycovirus 1 (BpPmV1)*, the mycovirus-containing strains were hypovirulent towards the experimental insects *Galleria mellonera* and physical and biochemical changes were observed in pore sizes, isoelectric point, and Pr1 activity. More notably, the mycovirus infection confers a significant environmental, abiotic, and biotic stress tolerance to the fungus [38].

In addition, it was studied whether the fungus *Beauveria bassiana* presented tolerance to stress when subjected to ultraviolet B radiation (UV-B) and infected or not with the virus, and it was demonstrated that this fungus, when infected with the virus, shows improved tolerance to UV-B stress, with an increase in germination rate and growth index after being subjected to 1200 mWm UV-B rays. This was also demonstrated in the fungi *Cordyceps chanchua* and *Cryphonectria parasitica*, which presented greater tolerance to UV radiation when infected with viruses, possibly due to alterations in the synthesis of pigments responsible for UV-B protections. This demonstrates the importance of the effect of the mutualistic interactions between fungus and virus, which tends to favor the resistance of these organisms to environmental factors, and is positive for the biological control of pests [44,45].

Moreover, forest ecosystems suffer from transformations caused by invasive species due to the elimination of the ecological function of tree species, problems that may be increased by the actions of climate change [43,44]. It is important to notice that insect pests, as well as plant pathogens and weeds, have negative effects on crops; these issues are increasing due to weather conditions [43,44].

### 3.4. Mechanism of Transmission of Mycoviruses

Mycovirus infections are usually asymptomatic, with few effects on host phenotype. Viral infection has been associated with alterations in certain phenotypic characteristics in their fungal hosts [33,34,35,36,37]. Mycoviral propagation can occur vertically through spores or horizontally through hyphal anastomosis [5,33,34,35]. It is transmitted intracellularly during cell division, sporogenesis, and/or cell-to-cell fusion; thus, virus life cycles generally lack an extracellular phase. Mycoviruses are mostly RNA viruses [36,37].

Buivydaite et al. (2024) [16] highlight the non-persistent vector-borne transmission of viruses. Viruses are limited to the mouth of the vector, enabling short-term transmission, and are not able to replicate in the vector. In contrast, in persistent vector transmission, also referred to as circulative transmission, viruses can cross the intestinal barrier of insects or arthropods and can be retained in the vector for extended periods or even replicate within the vector, facilitating long-term transmission. The authors propose the above hypothesis because the vector-borne transmission of mycoviruses could involve a similar mechanism given the diversity of natural fungivores that feed on fungi. Nevertheless, given that a significant portion of mycovirus genes have unknown function, some of them may serve as yet-unidentified vector transmission-associated proteins, requiring further investigation.

Ozkan and Coutts (2015) [34] conducted studies with *Aspergillus fumigatus*, a fungal pathogen that causes lung disease in humans and animals and is responsible for causing invasive aspergillosis in immunocompromised patients and chronic lung disease, and is also capable of causing allergic reactions in immunocompromised patients. These researchers used *Galleria mellonella wax* moth larvae to evaluate the pathogenicity of the fungus *A. fumigatus.* These larvae have been used as organisms to evaluate the virulence of some pathogenic fungi and bacteria, as well as the effect of antimicrobials. *G. mellonella*, has been used to evaluate the pathogenicity of bacteria such as *Pseudomonas aeruginosa* and *Legionella pneumoniae*, as well as the yeasts *Cryptococcus neoformans* and *Candida albicans.* The effect of mycoviruses on the pathogenicity of *A fumigatus* was evaluated using the *G. mellonella* infection model, demonstrating, for the first time, the effect of a *Partitivirus* and an A78 virus [46]. *G. mellonella wax* moth larvae is a reliable and low-cost organism, can survive in a wide temperature range, is very easy to handle, and is easy to inject due to its size, and the infection has a short monitoring time [46,47,48].

Vector-mediated transmission of mycoviruses may involve arthropods, nematodes, and other animals that serve as intermediaries for virus transfer to new hosts. One example is the mycovirus *SsHADV1*, which is transmitted by the frugivorous insect *Lycoriella ingenua* [11]. This mycovirus follows a persistent transmission mode, replicating within the insect host and being vertically transmitted to its offspring via transovarial transmission. Another case involves the mite *Thyreophagus corticalis*, which feeds on chestnut canker. This arthropod is capable of transmitting *Cryphonectria hypovirus 1 (CHV-1)* to another *C. parasitica* strain through feces containing mycelia infected with *CHV-1*. This mechanism is considered indirect nonpersistent transmission via a vector. Additionally, mechanical transmissions have been described in which tissue injury leads to the release of virions, facilitating virus spread through mechanical forces, a process well-documented in soil-borne plant viruses and potentially applicable to mycoviruses. Currently, evidence on the vector-mediated transmission of mycoviruses remains scarce [18].

### 3.5. Field of Study of Mycoviruses

Although many mycoviruses do not have marked effects on their hosts, those that reduce the virulence of their phytopathogenic fungal hosts are of considerable interest for the development of novel biocontrol strategies [5]. As compared to other fields of virology, mycovirology is still in its infancy; further development may provide long-term, sustainable solutions to a range of pertinent health and environmental issues, including drug resistance, biocontrol of infectious diseases or arthropod, pests, food security, agriculture, and forestry [5]. Similarly, *Sclerotinia* fungal genera are considered plant pathogens that are responsible for stem rot, and many mycoviruses have been isolated from this genus, some of which have shown hypovirulence [16].

Furthermore, mycoviruses cause hypovirulence or attenuate pathogenicity in fungi, resulting in reduced conidiation and a decreased growth rate and pigmentation. Hypervirulence caused by mycoviruses in fungus leads to increased sporulation and increased growth [3].

Various methods have long been used in agriculture to avoid the problems caused by these fungi include crop rotation, fungicides, and sanitation, among others. This has generated resistance, and their residues have caused damage to the environment, farmers, and consumers. Some pesticides can remain in the soil for up to 10 years, posing a significant threat to the environment, and have also caused cancer, skin allergies, and other pathologies in animals and humans; thus, the use of microorganisms to control these crop diseases provides a more advanced, environmentally friendly, and safer method for farmers. Therefore, the use of mycoviruses as fungicides is an appropriate and promising method [16].

### 3.6. Mycoviruses as Strategies of Biocontrol

Biocontrol is an environmentally friendly alternative. Shi et al. (2019) [35] used Tobacco Mosaic Virus (TMV) to transport antifungal proteins into host plants cells to increase disease resistance [6]. The procedures involved in the use of mycoviruses as biocontrol will depend on the objectives and the target or pathogen to which they are directed. First, the mycovirus must be isolated and characterized: the fungus is cultured, and subsequently the viral particles are extracted and sequenced for identification. Then, the mycoviruses are biologically characterized to understand their mode of transmission and how they affect the host fungus. The host range is then evaluated using techniques such as co-culture, protoplast fusion, hyphal anastomosis, and the construction of infectious clones to extend this host range to constant infections. The effectiveness of the infection caused by the mycovirus in the infectious agent is determined analyzing the virulence, reproductive development, and growth of the mycoviruses to be used as a fungicide [1,44,45].

From an agricultural point of view, mycoviruses could contribute to the development of sustainable agriculture since they might act as biological control agents, causing changes in the physiology of fungi and decreasing their virulence [9].

It is important to highlight the example of the successful use of mycoviruses as a form of biocontrol of pathogens or phytopathogens in the chestnut blight in Europe. The *Cryphonectria hypovirus 1 (CHV-1)* virus was used against the fungus *Chryphonectria parasitica*, a fungus that causes the disease called “blight” in the plants. In this case, fungi infected with the hypervirus *C. parasitica* were placed on the margins of the cankers that appear on the bark. A favorable result was observed after the application of the hypovirus with a change in shape from sunken to swollen or the process of healing, increasing the probability that the trees survived. The disadvantage of this technique is that it is not viable to be used intensively and on a massive scale [43,49,50].

Another example of a mycovirus used as a biocontrol agent for plant pathogenic fungi, which could serve as an alternative to chemical control, is *Rosellinia necatrix Partitivirus 2*, a member of the *Alphapartitivirus* family. It infects the fungus *Rhizoctonia solani*, a soil *basidiomycetes*, causing infections in ornamental plants and tree species [3,4]. Another example is *Heterobasidion partitivirus*, which causes phenotypic weakened strains of fungi *H. ecrustosum*, *H. annosum*, and *H. parviporum*, species of fungi that cause root rot in forest plants [5].

## 4. Conclusions

Mycoviruses represent a novel field of study. Mycoviral transmission mechanisms can occur vertically or horizontally, and can be transmitted intracellularly and by cell-to-cell fusion, thus affecting their life cycles. The way mycoviruses are transmitted is persistent, which means that they replicate in the host insect and are transmitted to its offspring through infected eggs. Nonpersistent transmission may occur when the insect manages to transmit the virus to another strain through feces containing mycelia infected with the mycovirus. Mycoviruses have no extracellular route of transmission, except for a single DNA mycovirus with associated hypovirulence and when applied extracellularly, but they are capable of reducing virulence in several host fungi. Understanding the biology and ecology of mycoviruses is essential for advancing their potential use as a biocontrol of pathogens, including phytopathogens, and their role in enhancing the effects and survival of entomopathogens, representing a promising area of applied research.

## Figures and Tables

**Table 1:** Mycovirus: genetic material, fungal hosts, and transmission routes.

Mycovirus Family/Genus	Individual Mycovirus Species	Type of Virus Genetic Material (Positive RNA, dsRNA, DNA)	Fungal Host Species	Known Transmission Routes	Note	Bibliografía
Unclassified mycoviruses/Geminivirus	*Sclerotinia sclerotiorum hypovirus 1* *SsHADV-1*	circular ssDNA	*Sclerotinia sclerotiorum*	It uses mycophagous flies as a vector transmitter. It is transmitted vertically to offspring through eggs but also horizontally to uninfected fungal hosts.	It stimulates plants growth and improves resistance to infection in pathogenic fungi.	[1,29]
*Alphaflexiviridae/Sclerodarnavirus*	*SsDRV* *Sclerotinia sclerotiorum RNA virus L*	+RNA	*Sclerotinia sclerotiorum*	Transmitted inconsistently transmitted via ascospores.	Appears to be related to hepatitis E and rubi-like viruses.	[29]
Alphaflexiviridae/Botrexvirus	*Botrytis virus X*	+RNA	*Botrytis cinerea*	Set of genes involved in cell-to-cell and long-distance movements of the plant-infecting members of this family.	It is capable of promoting distinct hypovirulence in its phytopathogenic fungal hosts.	[27,29]
*Gammaflexiviridae/Mycoflexivirus*	*Botrytis virus F*	+RNA	*Botrytis cinerea*	Is also present in ascospores.	It is capable of promoting distinct hypovirulence in its phytopathogenic fungal hosts.	[29]
*Hypoviridae/Hypovirus*	*Sclerotinia sclerotiorum hypovirus 1 and 2 (SsHV1/SsHV1/SZ-150)*	dsRNA/+RNA	*Sclerotinia sclerotiorum*	The sclerotia produced by *S. sclerotiorum* might play an important role in the vertical transmission of mycovirus in this fungal species lacking conidia.	It is capable of promoting distinct hypovirulence in its phytopathogenic fungal hosts.	[2,29]
*Hypoviridae/Hypovirus*	*Valsa ceratosperma hypovirus 1 (VcHV1)*	dsRNA	*Valsa ceratosperma*	Infection occurs via hyphal anastomosis.	It is capable of promoting distinct hypovirulence in its phytopathogenic fungal hosts.	[2,28,29]
*Hypoviridae/Hypovirus*	*Cryphonectria Hypovirus 1* to *4* *CHV1* *CHV2* *CHV3* *CHV4*	mcRNA (+)/dsRNA	*Cryphonectria parasitica*	Infection of fungal mycelium is known to occur only through hyphal contact or horizontal transmission.	Used as biological control for chestnut blight in Europe.	[2,4,34,35]
*Hypoviridae/Hypovirus*	*Fusarium graminearum Hypovirus 1 FgV1 y FgV4. FgV2*	+RNA	*Fusarium graminearum*	Anastomosis hyphal.	It is capable of promoting distinct hypovirulence in its phytopathogenic fungal hosts.	[2,4,29,36]
*Partiviridae/Betapartivirus*	*Betapartitivirus Fusarium poae virus 1*	dsRNA	*Fusarium poae*	Anastomosis hyphal, cell division, and sporogenesis in fungi.	It is capable of promoting distinct hypovirulence in its phytopathogenic fungal hosts	[4,7,9]
*Partiviridae/Betapartivirus*	*Sclerotinia sclerotiorum partitivirus 1*	dsRNA	*Sclerotinia sclerotiorum*	Horizontal transmission.	It is capable of promoting distinct hypovirulence in its phytopathogenic fungal hosts.	[3,29]
*Partiviridae/Gammapartivirus*	*Penicillium stoloniferum virus F*	dsRNA	*Penicillium stolonifer*	Hyphal anastomosis, cell division, and sporogenesis in the fungus.	It is capable of promoting distinct hypovirulence in its phytopathogenic fungal hosts.	[4,7,29]
*Partiviridae/Gammapartivirus*	*Penicillium stoloniferum virus S*	dsRNA	*Penicillium stolonifer*	Hyphal anastomosis, cell division, and sporogenesis in fungi.	It is capable of promoting distinct hypovirulence in its phytopathogenic fungal hosts.	[4,29,37]
*Partiviridae/Partivirus*	*HetPV3-and1*	dsRNA	*Heterobasidion parviporum*	Horizontal transmission via basidiospores.	Some are conifer pathogens. Used as biological control.	[2]
*Quadriviridae/Quadrivirus*	*Rosellinia necatrix quadrivirus 1*	dsRNA	*Rosellinia necatrix*	Infected mycelia.	Virocontrol.	[29]
*Megabirnaviridae/Megavirus*	*Megabirnavirus 1 (RnMBV1)*	dsRNA	*Rosellinia necatrix*	Used a zinc-mediated method for horizontal transmission of viruses between mycelia.	Potential biological control of white root rot (white root rot fungus is destructive to many crops, particularly perennial fruit trees).	[2,38]
Reoviridae/Reovirus	*Myco Reovirus*	dsRNA	*Sclerotinia sclerotiorum*	Vertical transmission via conidia. Horizontal transmission.	Capable of promoting distinct hypovirulence in their phytopathogenic fungal hosts.	[2]
	*MyRV1* *MyRV2*		*Cryphonectria parasitica*			[29]
	*MyRV3*		*Rosellinia necatrix*			[3]
*Barnaviridae/Barnavirus*	*Mushroom bacilliform virus (MBV)*	dsRNA	*Agaricus bisporus*	Horizontal transmission via mycelium and possibly vertically by basidiospores.	Infects commonly cultivated button mushroom.	[2,5,29]
Totiviridae/Totivirus	*Saccharomyces cerevisiae virus L-A (ScV-L-A)*	dsRNA	*Saccharomyces cerevisiae*	Transmitted intracellularly.		[4,29]
*Totiviridae/Totivirus*	*Ustilago maydis virus H1* *Ustilago maydis virus P4*	dsRNA	*Ustilago maydis*	Transmitted intracellularly.		[4,29]
*Totiviridae/Victorivirus*	*Helminthosporium victoriae virus 190S (HvV190S)*	dSRNA	*Helminthosporium victoriae*	Transmitted intracellularly.	Virocontrol.	[4,29]
*Chrysoviridae/Chysovirus*	*Penicillium chrysogenum virus (PcV)*	dsRNA	*Penicillium chrysogenum*	It has been possible to induce a sexual cycle yielding meiotic ascospores. Can be transmitted intracellularly.	Progenies of *P. chrysogenum* are likely to be virus-free, thus potentially improving strain stability. Virocontrol.	[4,29]
*Chrysoviridae/Chrysovirus*	*Cryphonectria nitschke chrysoviridae 1*	dsRNA	*Cryphonectria nitschke*	Mycoviruses can be transmitted intracellularly.	Virocontrol.	[4,29]
*Narnaviridae/Narnavirus*	*Narnavirus*	+ssARN	*Saccharomyces cerevisiae and Phytophthora infestans*	Are confined to the cytosol.	Capable of promoting distinct hypovirulence.	[5,29]
*Mycomononegativiridae/*	*Sclerotinia sclerotiorum negative-stranded RNA virus 1 (SsNSRV1)*	−RNA	*Sclerotinia sclerotiorum*		Capable of promoting distinct hypovirulence.	[29]
*Virgaviridae/Viviviruses*	*PvLaVVV1* to *PvLaVVV4*	+ssRNA	*Aspergillus flavus*	Horizontal gene transfer between virus and host has been recognized as an important driving force in the viral evolution.	Marked differences reported in growth, spore production, and competitive ability between isogenically infected and uninfected *Aspergillus* strains.	[7,29]
*Ambivirus*	*Tulasnella ambivirus 1 (MN793991*; *4736 nt)*	ssRNA	*Cryphonectria parasitica*; *Rosellinia solani*; *Armillaria* spp.; *Helminthosporium parviporum*	Horizontal gene transfer between virus and host has been recognized as an important driving force in the viral evolution.	Morphological characterization is necessary.	[5]
*Yadokariviridae*	*Yado-kari virus 1 (YkV1)*	*+ssRNA*	*Rosellinia necatrix*	Horizontal gene transfer between virus and host has been recognized as an important driving force in the viral evolution.	Morphological characterization is necessary.	[5]
*Polymycoviridae/Polymycovirus*	*Aspergillus fumigatus tetramycovirus 1 (AfuPmV1)*	dsRNA	*Aspergillus fumigatus*	Infected mycelia associated with one of the virally encoded proteins.	Morphological characterization of *polymycoviruses* is needed.	[5]
*Polymycoviridae/Polymycovirus*	*Colletotrichum camelliae filamentous virus 1 (CcFV1)*	dsRNA	*Colletotrichum camelliae*	Vertical (sexual spores) and horizontal (hyphae anastomosis) transfer of the virus.	Does not cause hypovirulence.	[5,7]

The most studied families of mycoviruses, including their genus and species, host fungi, genetic material, transmission routes, and the most notable characteristics, can be found in the bibliography.
